# Management of Severely Reduced Interimplant Distance Using a Novel Eccentric Abutment: A 6‐Year Case Report Featuring Digital Quantification of Implant Spacing

**DOI:** 10.1155/crid/9428747

**Published:** 2026-04-21

**Authors:** David Morales Schwarz, Jorge Manuel Perez Taveras, Serge Szmukler-Moncler, Ece Atay, Florian Beuer

**Affiliations:** ^1^ M&M Clinica Dental, C. Estadio 1, Valladolid, 47006, Spain; ^2^ Department of Prosthodontics, Charité University of Medicine, Charité Center 03, Assmannshauser Str. 4-6, Berlin, 14197, Germany, charite.de; ^3^ Berlin Implantology Research Group, Eichhornstrasse 2, Berlin, 10785, Germany

**Keywords:** conical connection, crestal bone preservation, digital computation, eccentric abutment, full-arch prosthesis, immediate loading of final prosthesis, interimplant distance, laser-welded framework

## Abstract

**Background:**

A minimum interimplant distance (IID) of 3 mm has been recommended to preserve the interproximal bone crest and soft tissue papilla. In immediate full‐arch rehabilitations requiring tilted posterior implants, anatomical constraints may not allow such spacing. When implants are placed in close proximity, conventional angulated multiunit abutments (MUAs) can lead to prosthetic interference and compromised mechanical resistance. This case report describes the management of two tilted implants with an IID <1 mm using a novel eccentric MUA. Implant spacing was precisely quantified using a superimposition‐based digital workflow.

**Case Presentation:**

A 72‐year‐old woman underwent extraction and immediate placement of six implants in the maxilla for full‐arch rehabilitation. Two posterior maxillary implants were positioned less than 1 mm apart. This prevented the use of conventional angulated MUAs. An eccentric MUA providing a 3.6 mm horizontal offset enabled restoration of adequate prosthetic spacing. A definitive prosthesis with a titanium laser‐welded framework and composite veneering was fabricated and delivered 24 h after surgery. At the 6‐year follow‐up, no biological or major mechanical complications were observed. In the narrow interimplant area, bone extended above both implant necks and the papilla persisted. After unscrewing the prosthesis, digital intraoral scanning with scan bodies and STL superimposition allowed digital measurements of the IID.

**Conclusions:**

This 6‐year case report documents the use of a noncustomized eccentric MUA with a 3.6 mm offset as a practical solution for managing an IID <1 mm. This was achieved without compromising prosthetic function or papilla formation. The crestal bone between the close implants appeared to remain stable over the follow‐up period. It is speculated that it may be related to the platform‐switching design and stability of the internal conical connection. Further clinical studies are warranted to assess the biomechanical reliability of eccentric abutments and the long‐term behavior of interimplant crestal bone when spacing is <1 mm.

## 1. Introduction

Full‐arch rehabilitation of the severely atrophic maxilla often relies on a combination of anterior axial implants and posterior tilted implants to avoid sinus grafting and engage the available bone envelope [1, 2]. In such configurations, interimplant distance (IID) may fall below the recommended 3 mm threshold [3–6], occasionally reaching values of 1–2 mm [7, 8]. This reduced spacing may compromise peri‐implant tissue maintenance and create prosthetic challenges.

Multiunit abutments (MUAs) are essential in immediate full‐arch protocols; they correct implant angulation and provide a standardized restorative platform [9, 10]. However, when implants are placed in close proximity, conventional MUAs may interfere with each other; they limit prosthetic space and complicate framework design, material thickness, and hygiene access.

An eccentric MUA, providing a horizontal offset of the prosthetic platform, offers a potential solution by laterally displacing the restorative interface without altering implant position. This approach may restore adequate interabutment spacing, facilitate prosthetic design, and improve soft tissue conditions.

This case report describes the use of a non‐customized eccentric MUA (Bioner, San Just Desvern, Spain) to manage two adjacent maxillary implants placed less than 1 mm apart in an immediate loading protocol, with a 6‐year clinical follow‐up and digital quantification of IID.

## 2. Case Presentation

Report of this case follows the CARE guidelines. The patient was treated according to the latest Declaration of Helsinki and an informed consent was signed to use her data under the conditions of anonymity.

### 2.1. Presentation of the Case

A 72‐year‐old female presented with the chief complaint of loss of retention of her maxillary removable prosthesis. She was aware of the critical state of her dentition and expressed the desire for a radical improvement of her dental condition.

Intraoral examination revealed a removable partial prosthesis replacing six anterior maxillary teeth, retained by metallic clasps on the first premolars (Figure 1a,b). The prosthesis relied on the roots of the anterior teeth; upon removal, these roots exhibited extensive caries associated with poor oral hygiene. The posterior right quadrant was restored with a three‐unit tooth‐supported bridge; the maxillary second molar showed mesial and distal amalgam restorations. The left posterior quadrant was rehabilitated with another three‐unit fixed dental prosthesis. The gingiva displayed generalized inflammation and recession; the latter were most pronounced in the right posterior region.

Figure 1Preoperative views: (a) with the maxillary prosthesis in place, (b) after removing it, and (c) panoramic radiograph taken after removal of the removable prostheses. Note the decay of anterior block and generalized periodontal loss.

**Figure:**
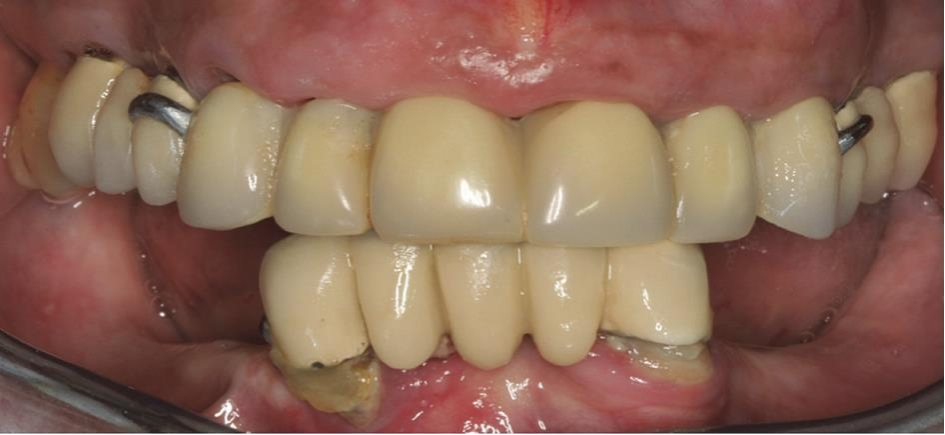
(a)

**Figure:**
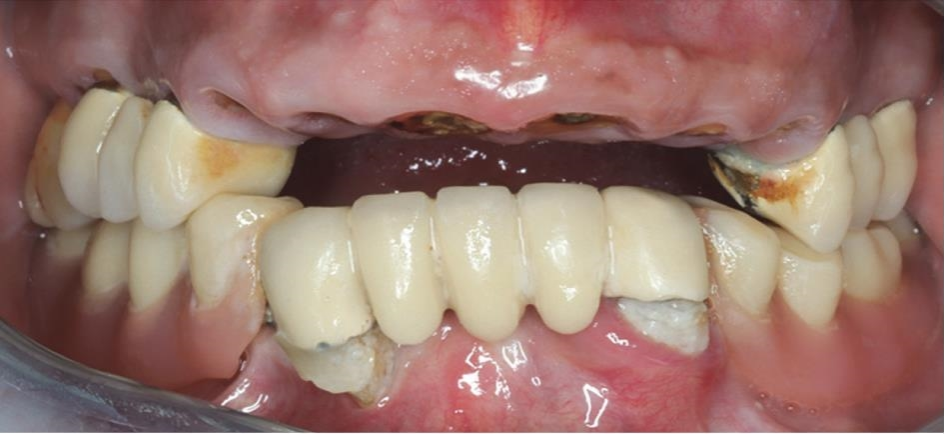
(b)

**Figure:**
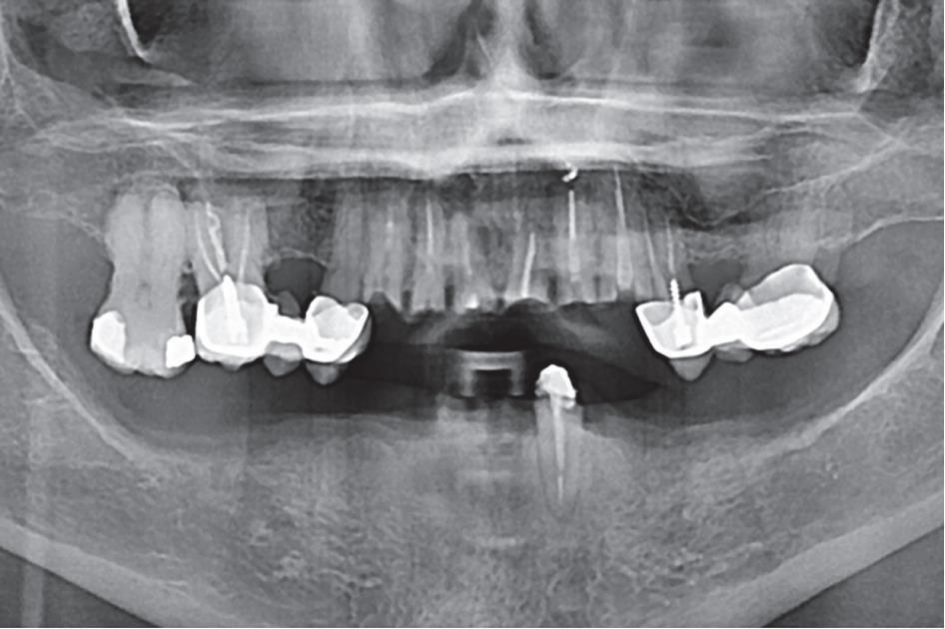
(c)

In the mandible, a five‐unit fixed prosthesis was supported by two natural teeth in the anterior region; both were covered with calculus deposits. The posterior segments were restored with removable partial dentures of four elements each (Figure 1a,b).

Radiographic examination (Figure 1c) confirmed the compromised condition of the maxillary anterior roots, generalized severe alveolar bone loss, and extrusion of the maxillary right second molar. In the mandible, carious lesions were noted beneath the metallic posts. Overall, the remaining teeth and roots were deemed hopeless due to advanced caries, periodontal breakdown, and poor hygiene.

The patient’s medical history revealed a smoking habit of 20 cigarettes per day. She had been under oral bisphosphonate therapy (Ácido Alendrónico, Sandoz 70 mg, Sandoz Farmacéutica SA, Madrid, Spain) for osteoporosis one tablet per week for 3 years; the treatment was discontinued 2 years earlier. Her systemic condition was classified as ASA II.

Clinical and radiographic evaluation included OPG and CBCT examinations; they confirmed severe coronal destruction and residual periapical pathology of the remaining teeth and pneumatized posterior sinuses.

### 2.2. Treatment Plan

Both the maxillary and mandibular arches were diagnosed as terminal dentition with a hopeless prognosis. A comprehensive bimaxillary implant‐supported rehabilitation was proposed.

The treatment plan included:

1. Extraction of all remaining maxillary teeth with thorough alveolar debridement, immediate placement of six implants in a hybrid configuration with two axial implants in the anterior region and two tilted posterior implants per side; the aim was to optimize anteroposterior spread and avoid sinus augmentation.
2. Immediate loading in the mandible and maxilla will be performed with a final screw‐retained prosthesis fabricated on a titanium framework [11–13], designed according to a digital wax‐up and produced by selective laser welding in the laboratory [14, 15]. The metallic framework will be first tried in the mouth to verify passivity before veneering with composite material.
3. In the maxilla, the prosthesis will be veneered with a composite layering system bonded to a metal substructure and subsequently characterized for esthetics.
4. In the mandible, the prosthesis will be fabricated with composite veneers integrated into an acrylic resin base to provide a more resilient occlusal interface with improved shock absorption while maintaining esthetic harmony between the arches.

Prior to treatment, blood tests included serum C‐terminal telopeptide (CTX) to assess bone turnover in the context of previous bisphosphonate therapy, thyroid‐stimulating hormone (TSH) to evaluate thyroid function and its potential influence on bone metabolism, and glycemia to exclude undiagnosed diabetes. These parameters were assessed to ensure appropriate systemic conditions for implant placement and osseointegration. The patient accepted the treatment plan after extensive explanation of the issues to address in both the mandible and maxilla. A written informed consent was signed that included the use of her data for scientific purposes under the condition of anonymity.

### 2.3. Treatment of the Case

Results of the blood sampling were the following:

– CTX was 0.15 ng/mL, below the 0.25 ng/mL threshold indicative of an effective antiresorptive response [16]. This level reflects suppressed bone turnover typically observed with long‐term bisphosphonate use and is associated with a low risk of MRONJ [17]. It does not contraindicate implant placement; however, it requires an atraumatic surgical protocol and careful planning.
– TSH was 3.22 mIU/mL, within the accepted reference range [18]. This finding indicates a borderline‐high euthyroid state without evidence of overt thyroid dysfunction. Bone metabolism and turnover were, therefore, considered compatible with predictable osseointegration.
– Glycemia was 76 mg/dL (<100 mg/dL), within normal limits [19].

### 2.4. Surgical Procedure

Under local anesthesia, all remaining maxillary teeth were extracted atraumatically; granulation tissue was removed and sockets were profusely irrigated with povidone–iodine betadine (Betadine Bucal 10 mg/mL, 200 mL, MedaPharma S.l, Madrid, Spain). Bone proved to be more brittle than usual, most probably because of the bisphosphonate treatment.

The maxilla was treated first; within the context of the present report focusing on two closely positioned tilted implants, only the maxillary procedure is described. A full‐thickness flap was raised, and implant placement followed an atraumatic surgical protocol adapted to the patient’s bone metabolic condition. The alveolar ridge was carefully leveled prior to implant insertion. Bone drilling was performed at a reduced rotational speed of 400 rpm [20], with abundant saline irrigation to minimize thermal and mechanical trauma.

Six TopDM implants (Bioner SA, San Just Desvern, Spain), made of titanium grade 23 with an inbuilt 0.25 mm platform‐switching feature and a hexagonally indexed 24° internal conical connection, were placed with a torque not exceeding 40 N cm to avoid excessive stress on the bone. The implant surface exhibited a regular pattern of macro‐ and micropores obtained by etching only (BioEtch), without sandblasting [21], extending to the collar and bevel. The implants were restored with angulated MUAs. Their positions were as follows:

• Anterior area, narrow axial implants of Ø 3.5 mm × 11.5 mm in Position 11 and Ø 3.5 mm × 7 mm in Position 21.
• Left posterior region, a tilted implant of Ø 4.0 mm × 13 mm emerging in Position 23 and another Ø 4.0 mm × 15 mm in Position 25; their angulation in mesial direction was superior to 30° to avoid the sinus.
• Right posterior region: a distal tilted implant (Ø 4.0 mm × 15 mm) was placed, emerging in Position 15 with a mesial angulation exceeding 30° to avoid the sinus. The adjacent mesial socket presented a large alveolar defect and poor bone quality; therefore, a second implant (Ø 4.0 mm × 13 mm) was placed in a tilted position within the limited area of healed bone, in close proximity to the distal implant. Consequently, the IID between the two tilted implants was well below the commonly recommended 3 mm threshold [3–6]. The implant neck was positioned approximately 2 mm more apically to achieve a primary stability of 35 N cm. This configuration was maintained to support the immediate loading protocol.
• As to the MUAs, implant in Position 11 received a straight one, four other implants, one in the anterior, and three in the posterior received 30° angulated MUAs. After seating an angulated MUA on the most distal right implant, the same could not be seated on the close mesial implant without infringing over the envelope of the MUAs; this prevented prosthetic rehabilitation (Figure 2a). To resolve this issue, an eccentric MUA (DM‐ECCA 15, Bioner SA, San Just Desvern, Spain) was fixed to the neck of the mesial tilted implant with a 25 N cm torque (Figure 2b,c). The shape of the eccentric MUA presents an offset of 3.6 mm between the center of the implant axis and the center of the conical head of the eccentric MUA (Figure 2c); in that way the distance between the external parts of the MUAs was increased. By shifting the prosthetic chimney away from the adjacent angulated MUA (Figure 2b,c) an additional room for titanium tube placement and composite bulk was created; in addition, it enlarged the soft‐tissue corridor between abutments.

After placing a protection cover on all MUAs, the residual gaps were filled with a particulate xenograft of deproteinized bovine bone mineral (DBBM; Creos Xenogain, NobelBiocare, Kloten, Switzerland; Figure 2d). A PRF membrane [22] was placed on the vestibular table to contain the xenograft particles (Figure 2d). Flap management was aimed to preserve periosteal vascularization and minimize tension during closure.

Figure 2Per‐operative dealing with the reduced IID. (a) Simulation of the placement of two standard angulated MUAs showing the impossibility to restore on these two close abutments (a1) and simulation with an eccentric MUA with a 3.6 mm offset (a2). These retrospective simulations were rendered possible after taking an intra‐oral scan at the 6‐year recall. (b) Per‐operative view with the eccentric abutment fixed to the mesial implant. Note the larger space for the titanium tubes of the restoration. (c) Drawing of the eccentric abutment showing an offset of 3.6 mm between the implant and the abutment axis. (d) Clinical view with all implants in place, the bone substitute filling and the PRF membrane.

**Figure:**
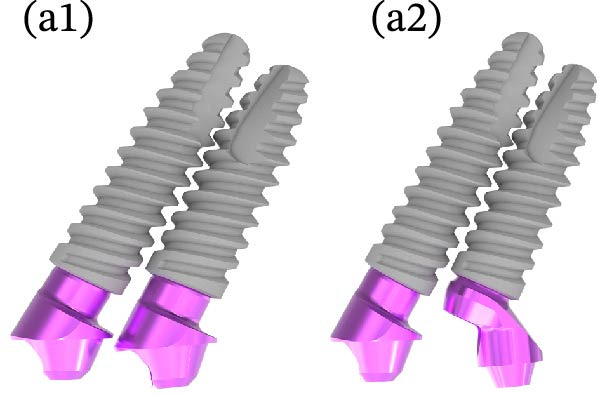
(a)

**Figure:**
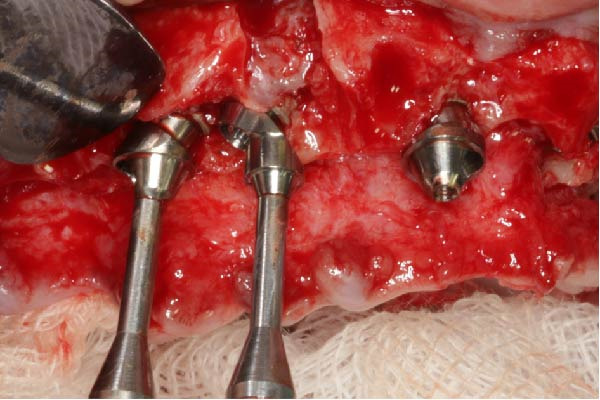
(b)

**Figure:**
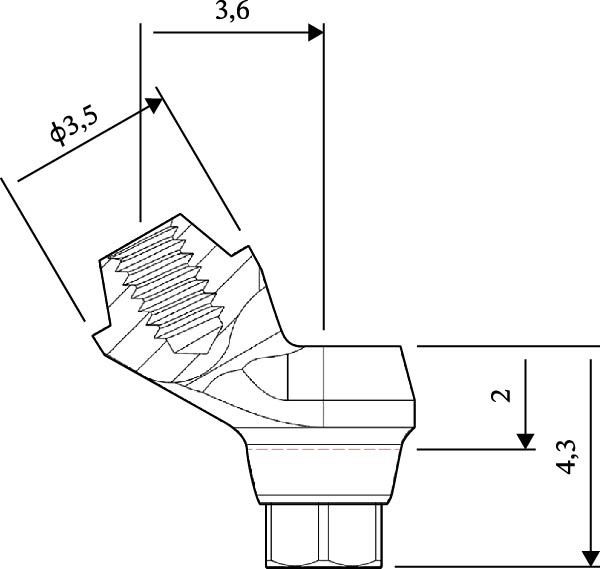
(c)

**Figure:**
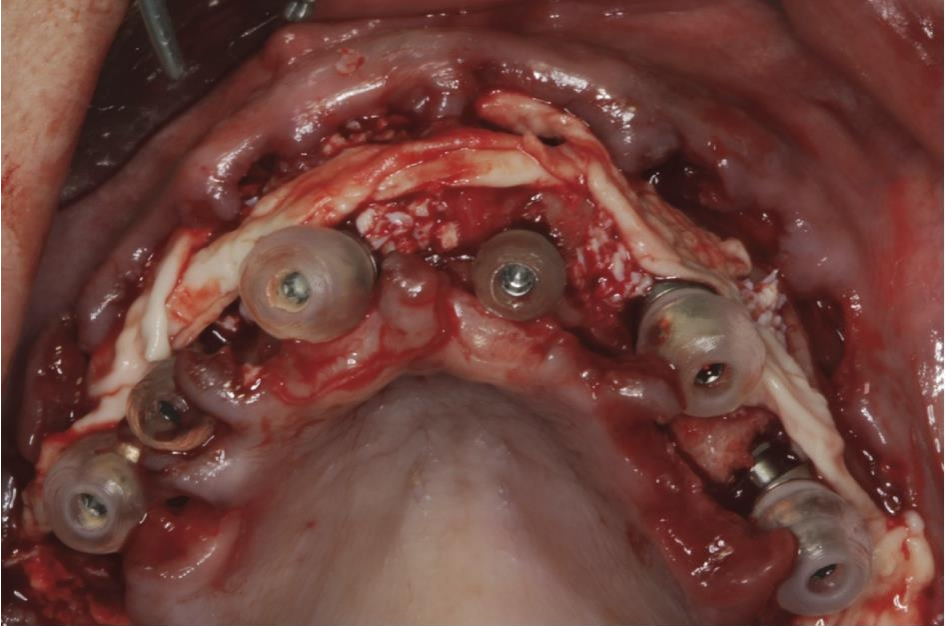
(d)

### 2.5. Prosthetic Procedure

Since all implants achieved a primary stability ≥35 N cm, the planned immediate loading protocol was implemented [23]. Impressions were taken at the MUA level using an open‐tray technique with splinted copings. A screw‐retained titanium framework with a tube‐in‐bar design was fabricated by laser welding. Passive fit was clinically and radiographically verified (Figure 3a,b), and a periapical radiograph confirmed a distance of approximately 1.2 mm between the neck of the distal implant and the eccentric abutment.

Figure 3Radiographic verification of the passivity of the titanium framework during the try‐in. (a) OPG of the try‐in of the two titanium frameworks. Passivity was obtained in the maxilla but not in the mandible. (b) Periapical radiography of the close implants. Note the effect of the eccentric MUA in creating a larger space between the titanium tubes of the metallic framework, the shape of the sinus, and the pristine bone levels that serve as baseline.

**Figure:**
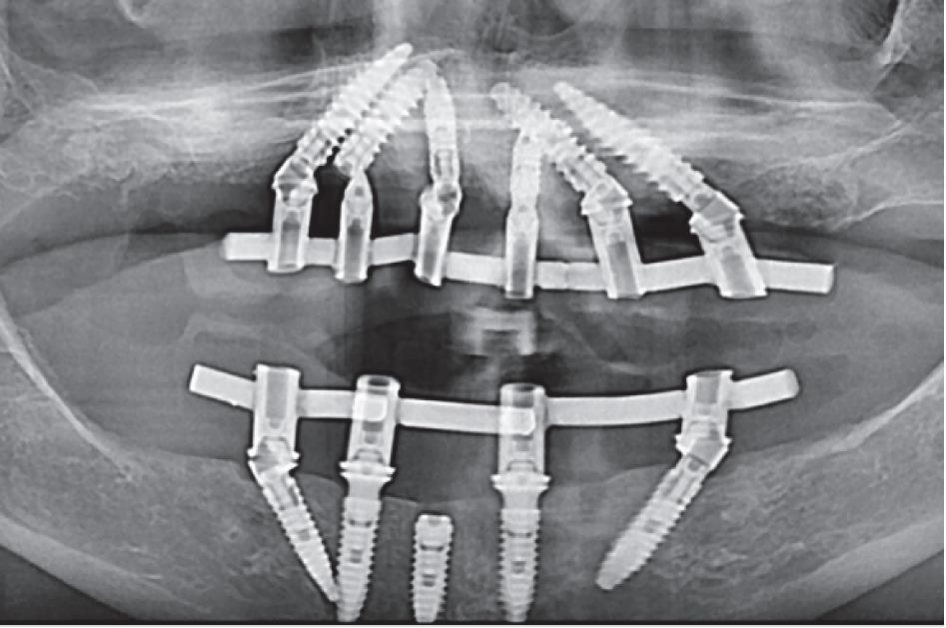
(a)

**Figure:**
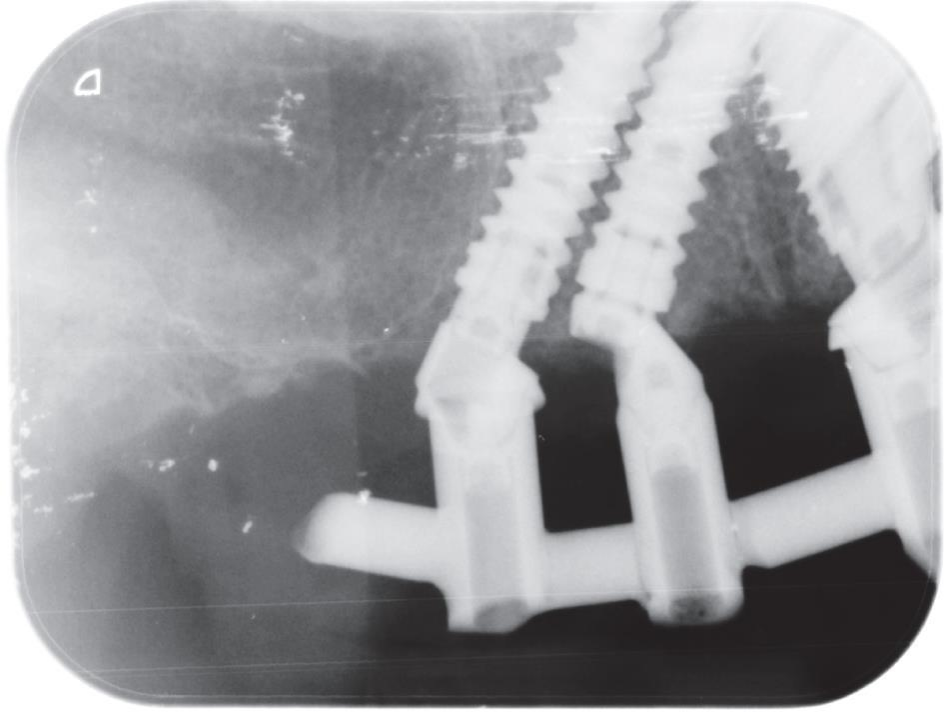
(b)

A definitive screw‐retained full‐arch prosthesis was then fabricated and delivered, incorporating composite veneering. The restoration included a cantilevered design adapted to the clinical situation (Figure 4a).

Figure 4Views of the definitive composite‐on‐titanium prosthesis. (a) Occlusal view showing the space between the chimneys of the close implants. Note the two‐unit premolarization cantilever on the right side and molar cantilever on the left side. (b) Frontal view of the upper and lower prostheses, each one with 12 crowns.

**Figure:**
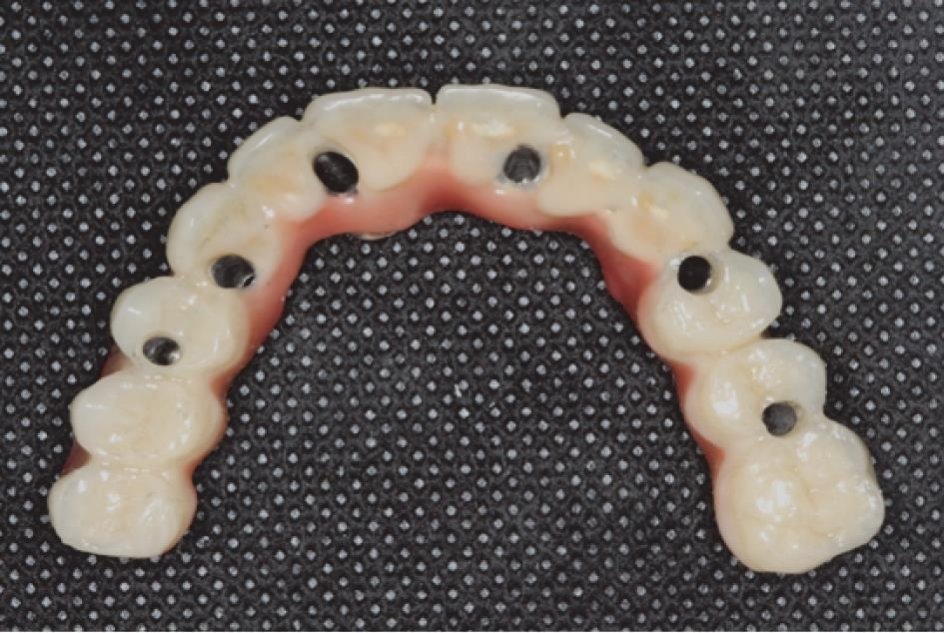
(a)

**Figure:**
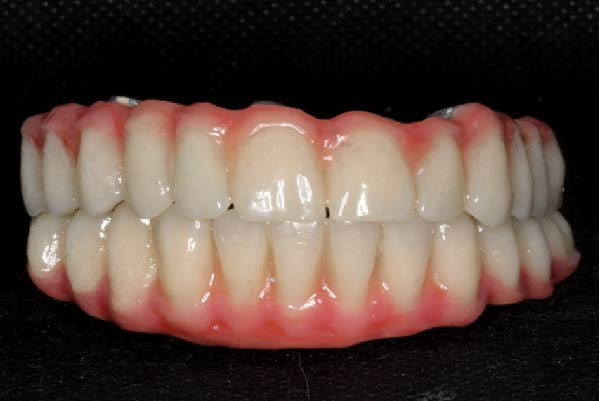
(b)

In the mandible, a similar screw‐retained prosthesis was delivered; it was supported by four implants as one implant could not be loaded (Figure 4b). The prosthetic design aimed to ensure functional stability, adequate esthetics, and a resilient occlusal scheme to optimize load distribution.

Twenty hours after surgery, the patient was rehabilitated with two final implant‐supported prostheses according to an extraction‐immediate implant‐immediate loading protocol (Figure 5a). After occlusal adjustments and an OPG radiographic control (Figure 5b) the patient was released.

Figure 5Delivery of the final prostheses 24 h after surgery. (a) Frontal view of the two prostheses. (b) OPG baseline control. Note the different materials used for the upper and lower prostheses in order to soften the occlusal stresses exerted on the implant‐supported rehabilitations.

**Figure:**
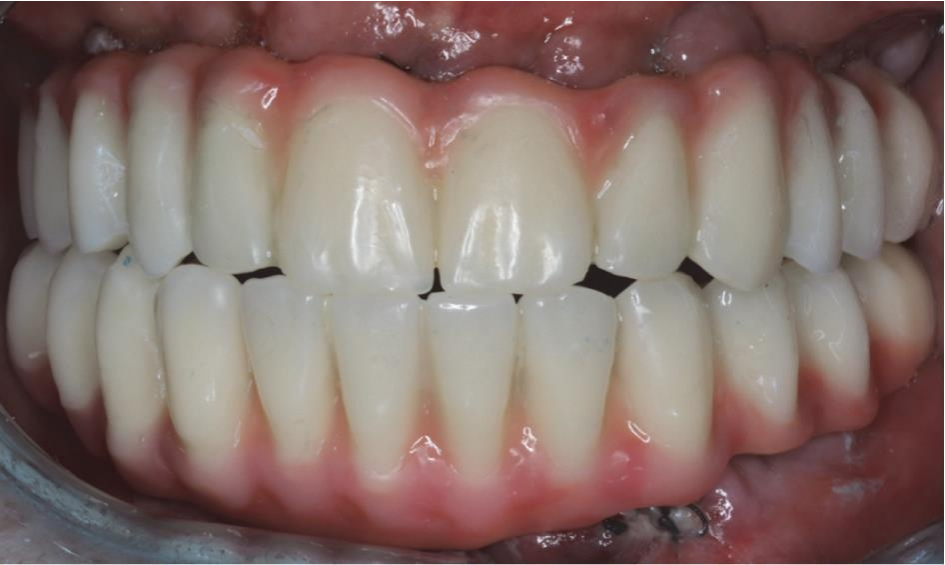
(a)

**Figure:**
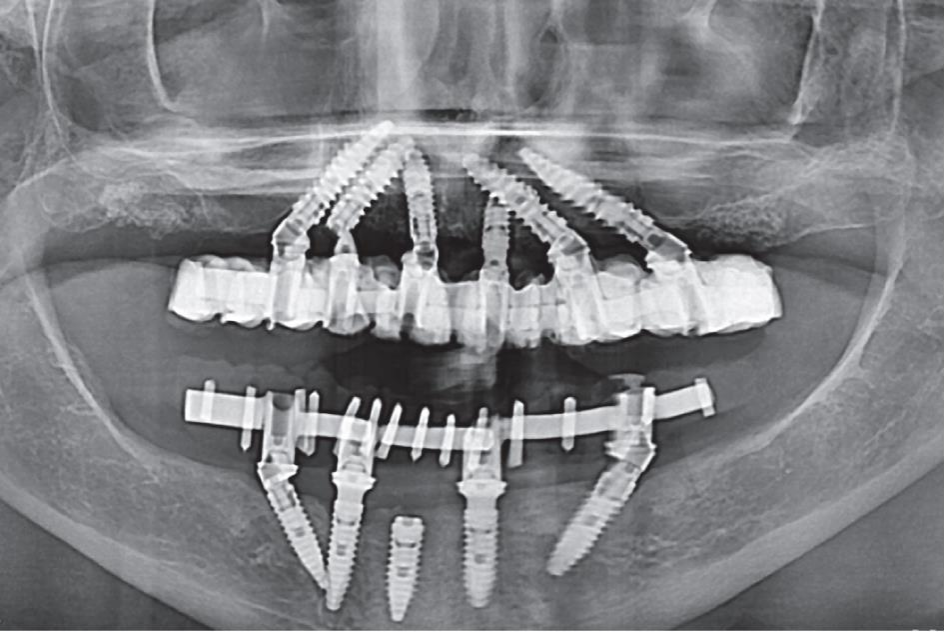
(b)

### 2.6. Postoperative Management

Postoperative management included antibiotic prophylaxis with amoxicillin, nonsteroidal anti‐inflammatory analgesics, and additional analgesics as needed. Oral hygiene instructions comprised twice‐daily chlorhexidine rinses for 1 week, along with a soft diet for 3 weeks and meticulous cleaning using interdental aids.

After 1 week, the patient was recalled for suture removal. Subsequent follow‐up appointments were scheduled at 1 month for clinical evaluation of soft tissue healing, at 3 months to assess prosthetic adaptation and include possible relining to improve its adaptation. Further evaluations were conducted at six and twelve months to monitor peri‐implant tissue health and patient comfort. The patient was then scheduled for recall every 6 months.

## 3. Follow‐Up of the Case

At the 3‐month follow‐up, the prostheses were unscrewed to assess implant stability and soft‐tissue healing. Figure 6a shows healthy and well‐shaped emergence profiles around all six maxillary implants. A close‐up view of the left posterior region (Figure 6b) highlights the pronounced papilla that developed thanks to the use of the eccentric abutment which redirected the emergence profile away from the distal angulated MUA.

Figure 6Soft tissue control 3 months after loading. (a) General view showing a healthy gingiva. (b) Close‐up view of the right posterior area. Note the presence of a large papilla thanks to the eccentric abutment that created enough space to be filled.

**Figure:**
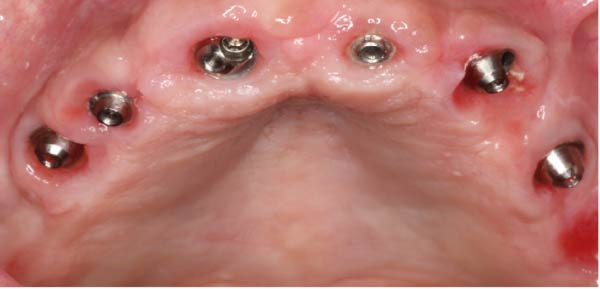
(a)

**Figure:**
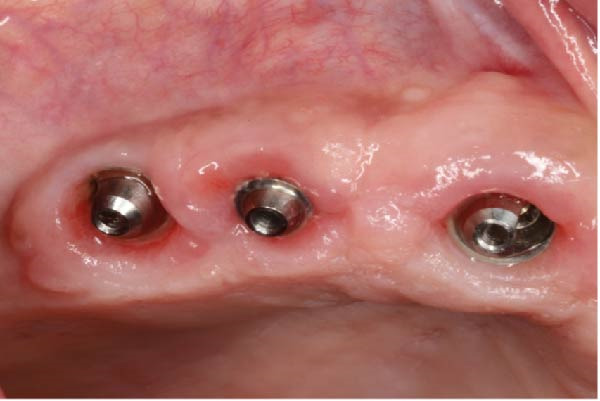
(b)

At each recall, the prosthesis was removed to clean the collected plaque caused by inevitable food accumulation. Observation of the successive OPGs did not reveal any notable bone loss around the implants; similarly, it could not provide an appropriate evaluation of the bony crest between the close implants. On the prosthetic level, chipping of crown #31 happened after 4 years and was immediately repaired.

At the 6‐year recall, the prosthesis showed discoloration consistent with heavy smoking (Figure 7a). The only notable radiographic finding was bone loss at the left tilted mandibular implant (Figure 7b). The mandibular prosthesis was then unscrewed, and clinical examination of the site revealed neither bleeding nor signs of suppuration.

Figure 7Six‐year control. (a) Frontal view with the prostheses in place. (b) Radiographic evaluation. In the maxilla, corticalisation around the coronal part of the implants was observed; it did not provide any information regarding the presence of bone between the close implants. In the mandible, bone loss was identified on the left tilted implants. (c) Periapical radiograph centered around the space between the adjacent implants. Note the presence of bone over the neck of the distal implant, surrounding the straight part of the angulated MUA and bone over the implant neck of the mesial one reaching the upper part of the eccentric MUA. (d) Inverse contrast of the periapical radiograph for a better reading of the bone delimitations. (e) Close‐up view of the soft tissue emergence of the two adjacent implants. The profile emergence displays a mild redness and plaque residues. Note a decrease, but no disappearance, of the papilla height as seen at 3 months.

**Figure:**
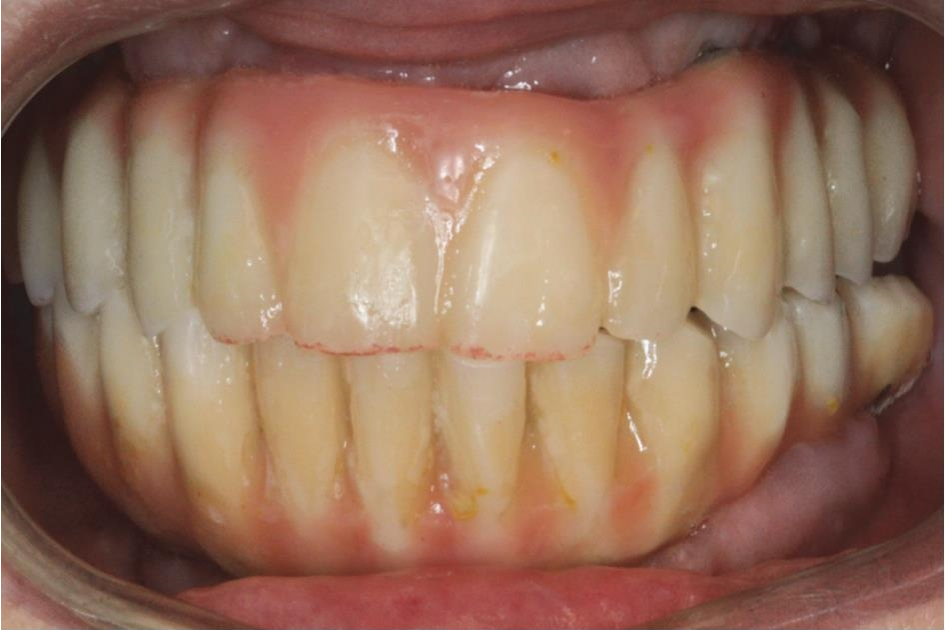
(a)

**Figure:**
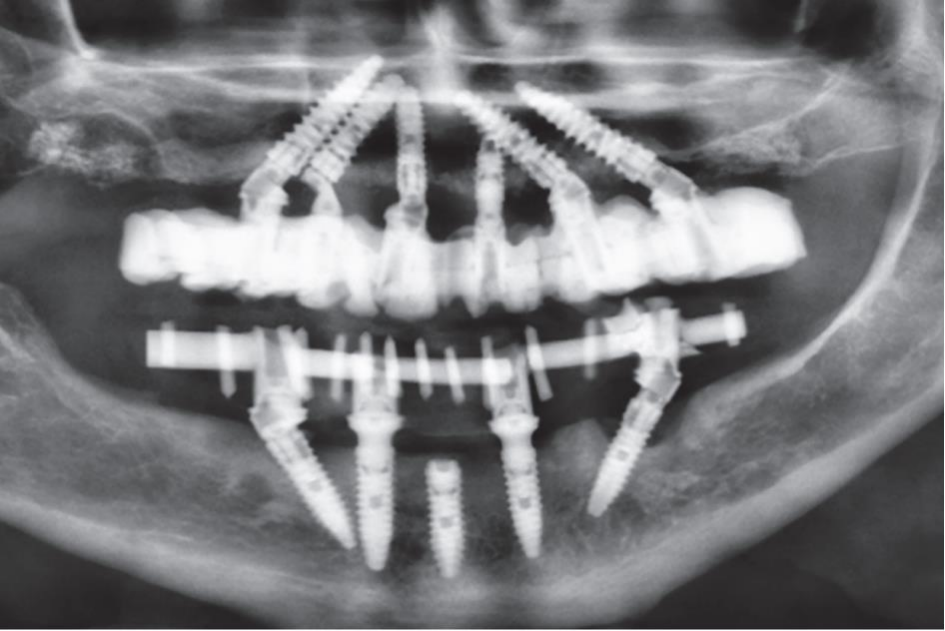
(b)

**Figure:**
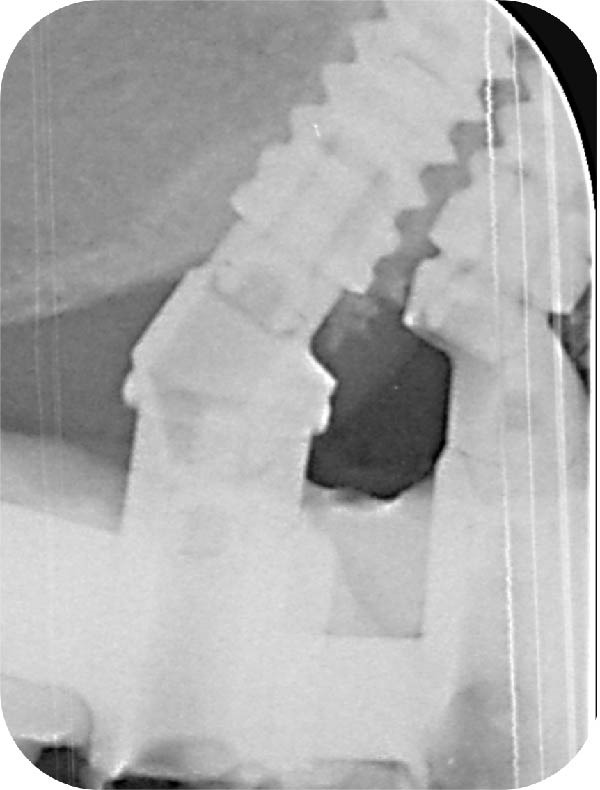
(c)

**Figure:**
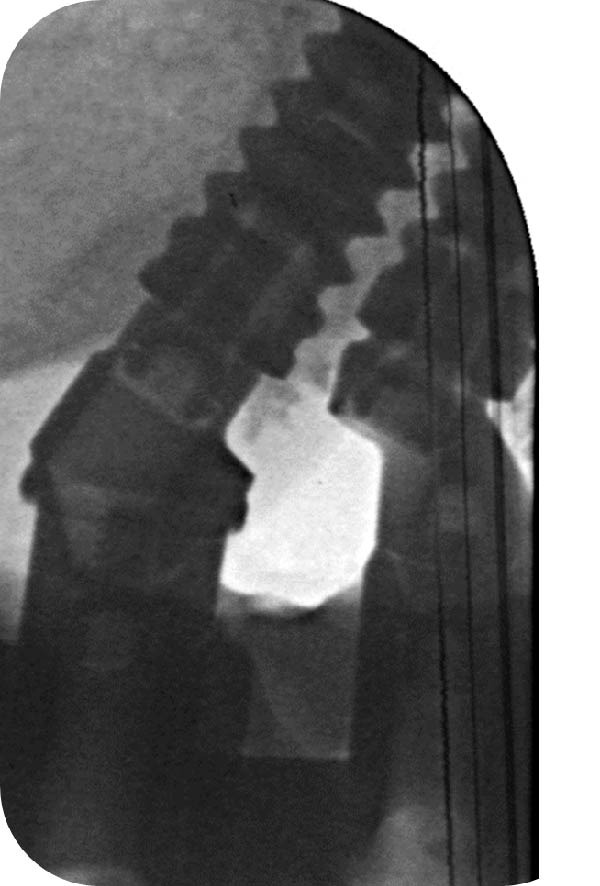
(d)

**Figure:**
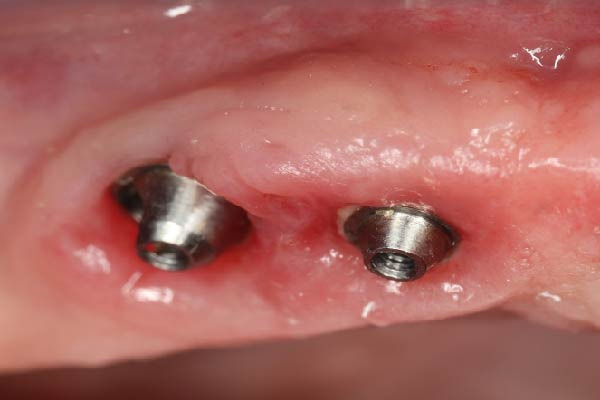
(e)

In the maxilla, given the initial concern about the limited IID of the adjacent implants of the left posterior area, a periapical radiograph was taken to assess the bone condition between the close implants (Figure 7c,d). Within the confined space between the implants, bone was present above both implant necks. On the mesial aspect of the distal implant, bone extended above the implant neck by 1.7 mm; it reached the straight portion of the angulated abutment. On the distal aspect of the mesial implant, bone was observed facing the upper portion of the eccentric abutment 2.4 mm above the implant collar. In addition, signs of bone densification were identified on the distal side of the distal implant, in contact with the straight portion of the angulated MUA above the implant neck.

The prosthesis was unscrewed to evaluate the peri‐implant soft tissue health. Some plaque and mild redness were consistently observed around the implants because of poor hygiene and plaque retention around the pontics. The interimplant papilla appeared reduced compared to the 3‐month control; it was, however, clearly present and well contoured (Figure 7e).

Periapical radiographs are conventionally used for IID assessment; however, inherent limitations of periapical imaging introduce a nonnegligible degree of measurement uncertainty, rendering such measurements approximate [24–26]. Accurate assessment, therefore, requires a measurement approach that avoids projection‐related distortion. For this purpose, the recent digital workflow proposed by Szmukler‐Moncler et al. [27] was implemented.

Scan bodies were affixed to the MUAs (Figure 8a) and an intraoral scan (Trios 3, 3Shape, Copenhagen, Denmark) was performed. For each implant, the STL files of the scan body, the MUA, and the corresponding implant were merged and superimposed (Exocad, Darmstadt, Germany and Meshmixer, Autodesk Inc., San Rafael, CA‐USA). This digital protocol allowed precise reconstruction of the three‐dimensional spatial relationship of the implants (Figure 8b,c) and accurate quantification of the IIDs on a plane section passing through the middle of the two adjacent implants.

Figure 8Digital determination of the various distances between the implants. (a) View of the scan bodies screwed on the standard and eccentric MUAs. (b) Computation of the 3D disposition of the close implants by successive superposition of the scan bodies (in white), the MUAs (in violet), the corresponding implants (in gray). (c) Other view of the adjacent implants showing the versatility of the 3D reconstruction through the intraoral scan with the scan bodies. (d) Measure of the various relevant distances separating the adjacent implants on a plane section passing through the middle of the two adjacent implants using the Meshmixer software. They are: distance between the neck of the distal implant and first contact with the eccentric abutment (1.44 mm), distance between the neck of the mesial implant and first contact with the distal implant (0.54 mm), distance of closest proximity between the two implants (0.14 mm), and the projected distance along a vertical line between the implant necks (0.23 mm).

**Figure:**
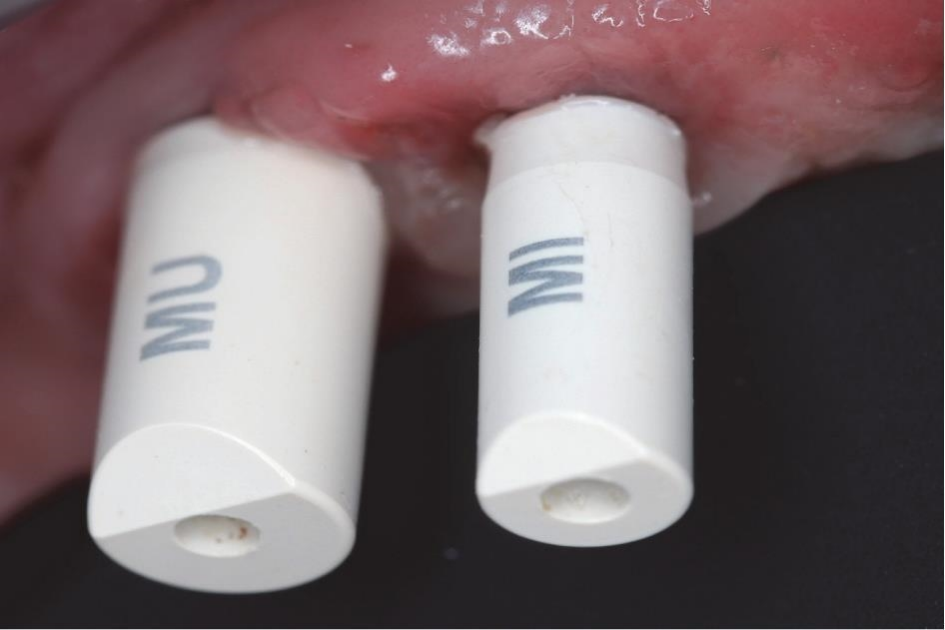
(a)

**Figure:**
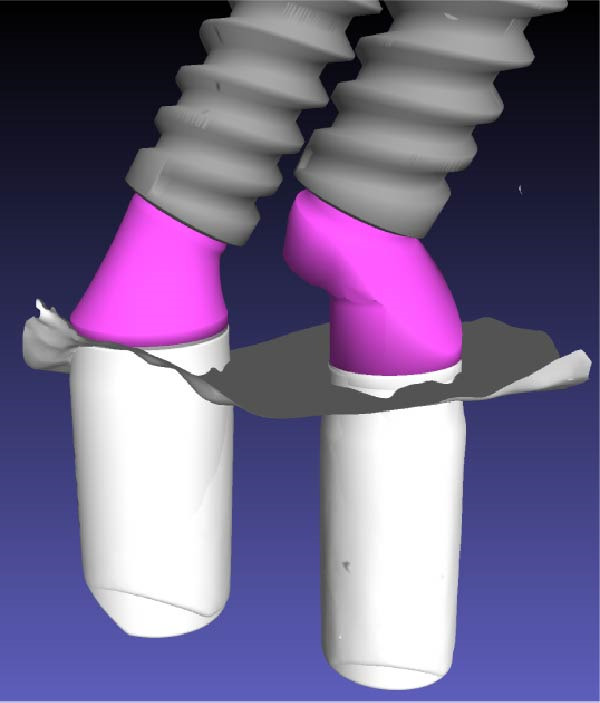
(b)

**Figure:**
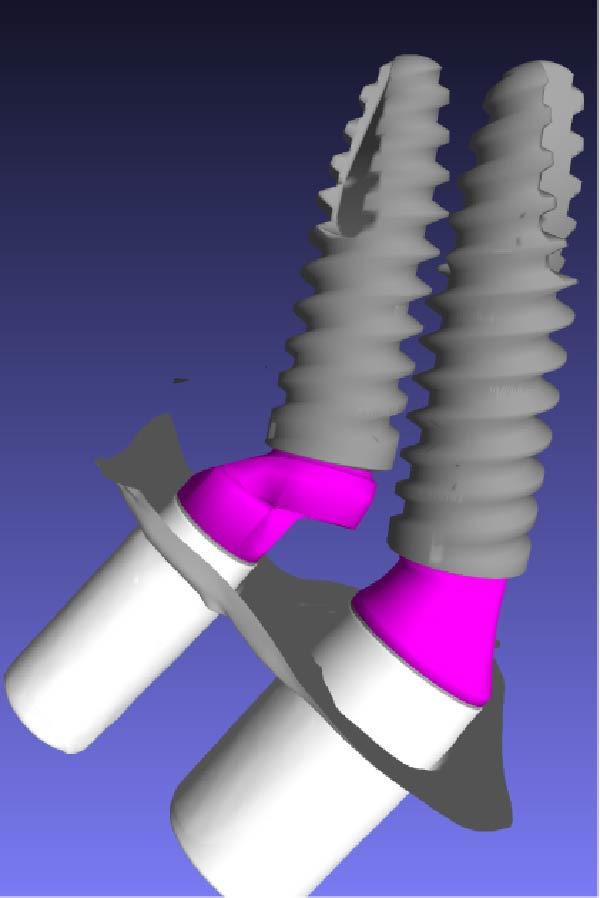
(c)

**Figure:**
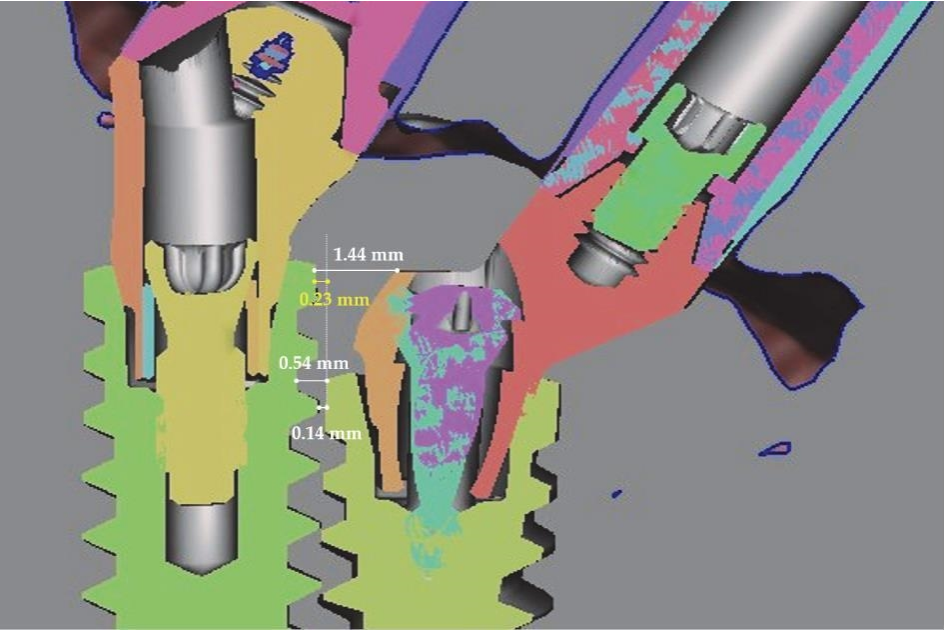
(d)

Due to the different coronoapical positions of the implants, four interimplant spacings were identified: between the neck of the distal implant and the eccentric abutment; between the neck of the mesial implant and the distal implant; at the point of closest proximity between the two implants; as the projected distance between the implant neck extremities. The respective distances were 1.44, 0.54, 0.14, and 0.23 mm (Figure 8d).

The minimum distance between the implants was 0.14 mm; however, this value does not correspond to the distance between the implant necks, which is the parameter most commonly used in the literature to define IID [3, 28, 29]. The projected distance between the implant neck extremities was 0.23 mm and was, therefore, considered to represent the clinically relevant IID.

The emergence profiles of all implants were carefully debrided to remove plaque deposits; the prosthesis was then reattached to the MUAs, and the patient was instructed to improve daily oral hygiene practices.

## 4. Discussion

This report describes the management of two adjacent implants placed with a severely reduced IID; this situation challenges both prosthetic feasibility and biological stability of peri‐implant tissues. While deviations from the recommended 3 mm IID [3–6] are not uncommon in daily practice [30–33], proximities of less than 1.5 mm remain rarely documented [8, 28, 29]. Such situations may arise when immediate‐placement protocols in adjacent extraction sockets constrain the available bone [7, 8], when discrepancies occur between digital planning and intraoperative execution [34, 35], or as a consequence of surgical inaccuracies [30–33]. The main clinical dilemma is whether to remove one implant or to maintain both and resolve the prosthetic constraints. In the present case, both implants were preserved in order to maintain the biomechanical integrity of the immediate loading protocol.

The main contribution of this report lies in the prosthetic solution. Conventional MUAs were not usable due to spatial interference. The use of a noncustomized eccentric abutment enabled a 3.6 mm lateral offset of the prosthetic platform; this feature restored adequate interabutment space without altering implant positions and allowed establishment of a soft tissue corridor conducive to papilla formation. The case highlights the use of a prefabricated eccentric MUA to resolve prosthetic interference between closely positioned implants; it provides a simple and reproducible alternative to more complex or customized solutions. To the best of our knowledge, such a prosthetic strategy has not been previously documented.

At 6 years, bone was maintained above the implant necks within the reduced interimplant space of 0.23 mm. These stable peri‐implant conditions may be related to the use of an in‐built platform‐switching design and a firm internal conical connection [8, 29]. The slight reduction in papilla height is likely related to hygiene limitations rather than implant proximity.

A definitive full‐arch prosthesis was delivered 24 h after surgery using a laser‐welded titanium framework fabricated in the laboratory. Although less frequently reported, several studies have documented the reliability of immediately loaded definitive prostheses [11–13, 36]. This approach shortens treatment time, avoids a provisional phase, and reduces costs and patient discomfort.

This report highlights the value of a digital approach using STL superimposition [27] for the precise assessment of IID. Periapical radiographs should be regarded as providing only approximate measurements, as they are inherently prone to geometric distortion and projection errors [24–26] and depend heavily on the alignment of the X‐ray beam relative to the implant vestibulopalatal and mesiodistal axes. In contrast, the digital STL–based measurements are more accurate and, therefore, served as the reference for assessing the various interimplant spacings defined in this report. Accordingly, the radiographic measurement (1.2 mm), taken between the implant neck and the abutment head, underestimated the actual distance determined by digital quantification (1.44 mm). This discrepancy is consistent with the known tendency of periapical radiography to underestimate measurements [33, 34].

This case report also addresses an important methodological aspect in the assessment of IID. When implants are not positioned at the same coronoapical level, a single linear measurement may not adequately describe their spatial relationship. In such situations, multiple distances can be identified depending on the level of measurement. The minimum distance between the implants was 0.14 mm and corresponded to the point of closest proximity. However, the projected distance of 0.23 mm between the implant necks was considered to better reflect the IID commonly reported in the literature addressing crestal bone stability [3, 28, 29].

In conclusion, the use of an eccentric MUA made it possible to complete the planned treatment and preserve both function and esthetics. While the present outcome was successful, it should be interpreted with caution, as it is based on a single case. Further studies are warranted to document the biomechanical behavior, long‐term stability, and biological safety of eccentric abutments.

## Author Contributions

Conceptualization, investigation, resources: David Morales Schwarz and Jorge Manuel Perez Taveras. Methodology: David Morales Schwarz, Jorge Manuel Perez Taveras, and Serge Szmukler‐Moncler. Formal analysis, writing – original draft: Jorge Manuel Perez Taveras and Serge Szmukler‐Moncler. Data curation, visualization: Jorge Manuel Perez Taveras, Serge Szmukler‐Moncler, and Ece Atay. Writing – review and editing: David Morales Schwarz and Florian Beuer. Supervision: Florian Beuer and Serge Szmukler‐Moncler.

## Funding

This research received no external funding.

## Disclosure

All authors have read and agreed to the published version of the manuscript.

## Ethics Statement

The study was conducted in accordance with the Declaration of Helsinki.

## Consent

Informed consent was obtained from the patient involved in this case report. Written informed consent has been obtained from the patient to use her data.

## Conflicts of Interest

David Morales Schwarz and Serge Szmukler‐Moncler declare that they receive consulting fees from the implant manufacturer. The other authors declare no conflicts of interest.

## Data Availability

The data that support the findings of this study are available upon request from the corresponding author. The data are not publicly available due to privacy or ethical restrictions.
